# Protein Association in Solution: Statistical Mechanical Modeling

**DOI:** 10.3390/biom13121703

**Published:** 2023-11-24

**Authors:** Vojko Vlachy, Yurij V. Kalyuzhnyi, Barbara Hribar-Lee, Ken A. Dill

**Affiliations:** 1 Faculty of Chemistry and Chemical Technology, University of Ljubljana, 1000 Ljubljana, Slovenia; barbara.hribar@fkkt.uni-lj.si; 2 Institute for Condensed Matter Physics NASU, 79011 Lviv, Ukraine; yukal@icmp.lviv.ua; 3 Laufer Center for Physical and Quantitative Biology, Stony Brook University, New York, NY 11794, USA; dill@laufercenter.org; 4 Department of Chemistry, Physics and Astronomy, Stony Brook University, New York, NY 11790, USA

**Keywords:** proteins, Wertheim’s theory, association, phase transition, antibodies

## Abstract

Protein molecules associate in solution, often in clusters beyond pairwise, leading to liquid phase separations and high viscosities. It is often impractical to study these multi-protein systems by atomistic computer simulations, particularly in multi-component solvents. Instead, their forces and states can be studied by liquid state statistical mechanics. However, past such approaches, such as the Derjaguin-Landau-Verwey-Overbeek (DLVO) theory, were limited to modeling proteins as spheres, and contained no microscopic structure–property relations. Recently, this limitation has been partly overcome by bringing the powerful Wertheim theory of associating molecules to bear on protein association equilibria. Here, we review these developments.

## 1. Introduction

Proteins can associate with each other in various non-covalent ways [1,2,3]. They can form dimers or multimers either specifically or nonspecifically, and in ways that are biologically functional or ways that are non-specific and non-functional. Protein associations can matter for disease states [4], such as amyloid formations in Alzheimer’s, Parkinson’s, and Huntington’s diseases. For reviews of the biological implications with an emphasis on the liquid-liquid phase separation see [5,6]. In addition, biotechnology requires developing appropriate formulations of protein molecules that result in stable folding and non-association [7]; these are often monoclonal antibodies (mAbs). The challenge is to formulate liquid solutions with a sufficient concentration of antibodies to be efficacious while at the same time not forming aggregates and not having high viscosity [8,9,10]. For these purposes, it is useful to have a microscopic understanding of the physical forces that drive proteins to associate.

## 2. Solution Experiments Provide Insights on the Driving Forces

**Figure 1:**
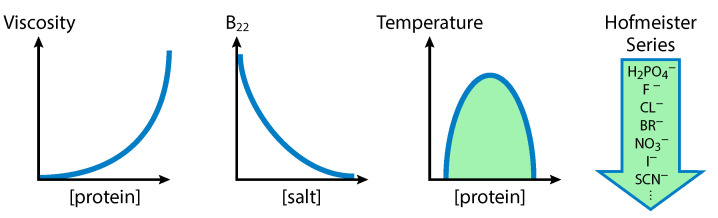
Driving forces of association as deduced from experiments. From left to right: proteins cluster beyond pairwise; electrostatics can be important; clustering is enthalpic; and hydration and hydrophobicity are important.

Figure 1 shows general observations about the driving forces behind association. From left to right the figure shows the (i) nonlinear increase of viscosity with protein concentration, suggesting that proteins self-associate in pairs or higher aggregates. (ii) Next panel shows that the second virial coefficient often decreases with increasing concentration of added salt, indicating that salt weakens the Coulomb repulsion of interacting proteins. (iii) Next one shows the liquid–liquid phase equilibrium, which can be observed to have an upper critical solution temperature, indicating an enthalpic driving force for protein clustering. (iv) The last panel suggests that there is an ordering of strength of effect by salt type corresponding to the Hofmeister series, which is an indication that hydration and hydrophobic interactions are important.

## 3. Background on Modeling the Forces in Protein Solutions

The mechanisms and driving forces of nonnative protein aggregation have been reviewed by Chi, Krishnan, Randolph, and Carpenter in [11,12]. They concluded that protein aggregation is controlled by conformational and colloidal stability and that either one of these can be rate-limiting depending on the solution conditions. Therefore, to control the protein self-association process, it is important to understand the factors that govern the self-association (pH, protein concentration, ionic strength, etc.) and the kinetics of the association/dissociation reaction [7]. Most existing studies, however, focus primarily on aggregation from only partial point of view, i.e., changes in conformational stability for isolated protein chains, protein–protein interactions between folded proteins, or changes in the binding propensities of hot spots [13]. Below, we provide a brief overview of the major milestones in understanding protein–protein interactions.

Two main approaches are used to study intermolecular forces at the microscopic level: atomistic computer simulations using semi-empirical force fields sampled by Molecular Dynamics (MD) or Monte Carlo (MC), and statistical mechanics-based liquid state theories. The advantages of MD modeling are its ability to capture atomistic-level details and its incorporation of motion dynamics [14,15,16,17,18,19,20,21,22,23]. Advances in simulations and machine learning emerging over the past decade [24] can help to reveal details at the level of the amino acids, and can guide drug discovery [25,26]. For a review of the computer simulation approach and how to identify aggregation-prone regions, see Agrawal et al. [27]. Further, within the last couple of years computer simulations using coarse-grained models have begun to serve as effective tools for interpreting experimentally observed data. Truskett’s group have used simple multi-bead models of monoclonal antibodies in MD simulations and combined them with scattering experiments to explain solution viscosities [28,29,30,31,32]. However, computer simulations are typically limited to relatively small proteins, small assemblies, or the early stages of formation [33,34,35].

In contrast, statistical mechanics-based liquid state models have been able to provide useful insights into more macro-scale behaviors such as liquid–liquid equilibria, viscosities, second virial coefficients, etc., though this incurs the loss of micro-scale atomistic details. Table 1 presents a brief history of statistical mechanical models and companion experiments. For a review of earlier work, see [36,37,38].

The traditional approach to predicting the thermodynamic properties of protein solutions is based on colloid theories such as the Derjaguin-Landau-Verwey-Overbeek (DLVO) theory [39,40]. While this theory has been proposed for lyophobic colloids, it has limitations when applied to proteins [41]. In most treatments, proteins are represented as hard spheres that interact through spherically symmetrical van der Waals and Coulomb interactions in salt water, using a continuum representation of the solvent and Debye–Hückel screening for salts. This is the so-called “colloidal approach”, the limitations of which have been discussed in [42,43]. DLVO often provides correct trends for the pH and salt concentration dependencies in protein solutions [44]; however, this approach does not readily account for protein sequence structure properties, explicit waters, or Hofmeister effects, where different salts have widely different powers of protein precipitation. A more subtle treatment is required to treat these effects [45]. In addition, the anisotropy is central to protein self assembly; thus, models with centrally symmetric interactions are poor representations of the non-spherical proteins in general and, due to their specific shape, antibodies in particular [46]. A number of refinements have been proposed to solve these problems, which are most notably connected with the J.M. Prausnitz, D. Bratko, B. Ninham, and W. Kunz, as well as many others (see for example [47,48,49,50], and see Table 1 as well).

Theoretical studies have been supported and further stimulated by significant experimental developments. One important finding was the discovery by George and Wilson [51,52,53] that the tendency of a protein molecule to crystallize can be predicted from the protein–protein second virial coefficient (B${}_{22}$). The importance of this discovery is that it allows a multi-protein action (crystallization) to be simplified through modeling, only requiring understanding of the first step, namely, the protein–protein pair interaction, measured by the second virial coefficient. Other binary coefficients, such as $k_{D}$, obtained from dynamic light scattering [54,55] and the Huggins viscosity coefficient [56], have proven to be very informative as well. For example, it has been shown that, as with B${}_{22}$, $k_{D}$ is correlated with the cloud point temperature, which determines the two-phase region in protein solutions [57].

Other key milestones were the experimental and theoretical liquid–liquid phase diagram studies of lysozyme and crystalline solutions by George Benedek and co-workers [4,46,58,59,60,61,62]. Of great relevance is the finding that protein folding stabilities are important for association. While reversible self-association of native proteins can occur at high enough concentrations, in most cases protein aggregation is a consequence of (partial) unfolding of the proteins [63,64,65]. These experimental findings stimulated further theoretical studies, in particular into liquid–liquid phase separations. These are discussed in more detail in the next paragraphs, together with the theoretical approaches designed to explain the measurements. Valuable information about protein aggregation has been obtained via viscosity measurements [8,10,66,67,68,69,70,71,72]. Knowledge about how to keep the viscosity of concentrated solutions low is important in the process of therapeutics production, formulation, and administration.

There are, however, several limitations of the early modeling approaches: (1) because proteins are treated as simple spheres with radially symmetric potentials, these approaches cannot capture the aspects of protein association that depend upon protein sequence and structure; (2) they provide inaccurate liquid–liquid phase diagrams, often predicting two-phase regions that are much narrower than those found in experiments (experimental phase diagrams often show broad flat tops around the upper critical solution temperature); and (3) they do not link the various macro-scale behaviors into a unified interpretation of phase equilibria, second virial coefficients, and viscosity. A general limitation of statistical mechanics-based liquid state theories is that they are low-order expansions, having a term for isolated monomers, then dimers, then trimers, etc. Such methods encounter challenges when handling the combinatorical complexity of increasingly larger aggregates.

Studying aggregation beyond these small aggregates requires approximations or coarse-graining, such as spherical particles or implicit solvents [20,23,34,73]. Thus, the MD approach is not practical for predicting phase diagrams, as a large number of proteins in a simulation box is required to assemble over large range of timescales. On the other hand, the group of Roberts et al. used coarse-grained multi-bead models in simulations to predict antibody–antibody interactions in solutions in a way that could account for experimental observations of the second virial coefficient [17,74,75,76,77]. Similar coarse-grained protein models have been used in statistical mechanical treatments, as described in the next section.

## 4. Statistical Mechanics-Based Treatments of Protein Interactions

**Figure 2:**
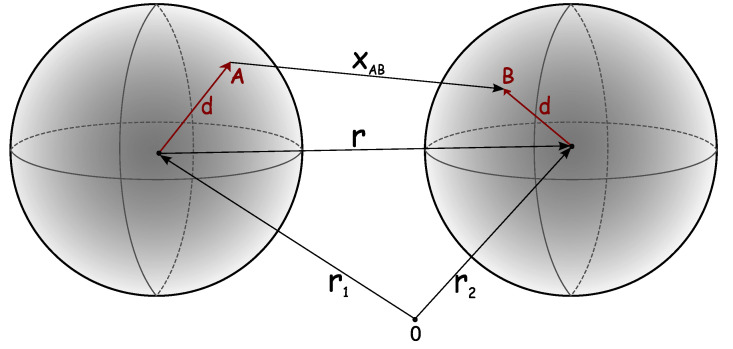
Model of the interactions of two globular proteins. Protein spheres interact at M × M pairs of binding sites on the surfaces, one pair of which (A and B) is indicated here. Reprinted by permission from [103].

Statistical mechanics-based liquid state models are able to provide useful insights into macro-scale behaviors such as liquid–liquid equilibria, viscosity, second virial coefficients, etc., however, this is achieved through the sacrifice of micro-scale details. Table 1 presents a brief history of statistical mechanical models and companion experiments. Following the seminal work of Sear [78], theories have been proposed based on the methods of condensed matter physics to predict the properties of “patchy colloids” as models for protein solutions [78,79,80,81,82,83,84,85,86,87,88,89,90,91,92,93,94]. Bianchi and co-workers [95,96] have shown that the width of the liquid–gas phase envelope crucially depends on the so-called “valency” of the colloidal particles, i.e., the maximum number of bonds the particle can form. These bonds are formed by the square well potential acting between off-center sites called patches; see Figure 2. As the valency decrease, the phase diagram width shrinks, and in the limit of two patches per particle the critical density approaches zero. The concept of the “empty liquid state” [95,96,97], i.e., the ability of a liquid to be stabilized at vanishingly low density, as well as the related concept of equilibrium gelation [98] formulated in these studies, appear to be very useful in biological applications [99,100,101,102]. By systematically varying the number of patches, it has been shown that there is strong correlation between the critical density and the valence of the particles [81]. In addition, it has been demonstrated that, for the appropriate number of patches, the theoretical and experimental phase diagrams for lysozyme and γ-crystallin as presented in the $T/T_{c}$ vs. $\rho /\rho_{c}$ coordinate frame are almost in quantitative agreement [81]. Here, $T_{c}$ and $\rho_{c}$ respectively indicate the critical temperature and the density.

For a review of studies published in the period before 2015, see [36,37]. Based on the previous studies mentioned above, our group has developed simple and computationally efficient models of protein–protein aggregation in salt solutions. They have been modeled as spherical objects, as dumb-bells, or as Y-shaped molecules, and decorated with binding sites that interact through the attractive square well potential of the depth and width in the range of the hydrogen bond values. Unlike simpler centrally symmetrical interaction models, the binding sites lead to orientation-dependent interactions between the proteins. Through several examples, we show how these models, in combination with Wertheim’s theories [104,105,106], can be helpful for analysis of measurements in protein solutions. This approach is complementary to the more detailed MD simulations.

## 5. The Wertheim Theory Treats Multi-Body Strong and Specific Associations

Around 2010, new theoretical approaches emerged to remedy deficiencies in protein solution modeling. Our group turned to the theory developed by Michael Wertheim in 1986, which is able to deal with solutions of particles that are non-spherical and have directional attractions [104,105]. The Wertheim theory would become the basis of SAFT (Statistical Associating Fluid Theory) for complex fluids, now widely used in chemical engineering [106,107,108]. A good pedagogical description of Wertheim’s theory can be found in [109]; here, we provide only a brief overview.

Protein association and clustering in solutions is caused by strong attractions between the particles; thus, many-body clusters are prevalent. Traditional statistical mechanics-based liquid state theories cannot handle these situations very well, as such theories are closely related to the Mayer expansion, which is formulated in terms of a single density. Efficient description of strongly associating fluids requires application of the theory based on the Mayer expansion formulated in terms of multiple densities (the so-called multidensity Mayer expansion) to represent cluster formation. Here, we first illustrate through a simple example that correctly reproducing the low-density limiting behaviors of strongly associating fluids requires an infinite number of terms in single-density Mayer expansions [110].

Considering the virial expansion for the pressure, we start with the expansions for the pressure P=P(z) and density ρ=ρ(z) in terms of the activity *z*, which in the low-density limit can be terminated at the second order to provide

(1) $$\beta P=z+b_{2}z^{2};\;\;\;\;\rho =z\frac{𝜕(\beta P)}{𝜕z}=z+2b_{2}z^{2},$$

where $b_{2}$ is the second virial coefficient, $\beta =1/k_{B}T$, $k_{B}$ is the Boltzmann constant, and *T* is the absolute temperature. Eliminating *z* between these two equations results in P=P(ρ), that is, the dependence of the pressure on the density:

(2) $$\frac{\beta P}{\rho }=\frac{1}{2}+\frac{\sqrt{1+8b_{2}\rho }-1}{8b_{2}\rho }.$$

At the same time, we can obtain P=P(ρ) from the conventional virial equation of state, which follows from (2) upon expansion of the square root with respect to the density, i.e.,

(3) $$\frac{\beta P}{\rho }=1-b_{2}\rho +4b_{2}^{2}\rho^{2}-20b_{2}^{3}\rho^{3}+\ldots .$$

For fluids having weak interactions between the particles, $b_{2}\to 0$ and both equations of state reduce (as they should) to the ideal gas equation of state, βP/ρ=1; however, a problem arises when treating the strong interaction limit, say, in the case of dimers. The correct equation of state for an ideal gas of dimers is βP/ρ=1/2 (a fluid with infinitely strong attraction between the monomers, i.e., $b_{2}\to \infty$), as follows from Equation (2); however, this result cannot be reproduced using expression (3) with a finite number of terms. Thus, for strongly interacting molecules in fluids, the correct and efficient description of the low-density behavior requires activity expansions [111,112].

The theory developed by Wertheim is based on combination of the density and activity expansions. This combination leads to a description of the equilibrium properties of the system in terms of the densities of the particles in different bonding states. To illustrate the theory, we briefly discuss the two-density version of the first-order Thermodynamic Perturbation Theory (TPT) formulated for a simple model of a dimerizing fluid. The model is represented by a hard-sphere fluid with an off-center attractive square-well (associative) site located on the surface of each particle (see Figure 2). The pairwise potential of the model U(12) and corresponding Mayer function f(12)=exp[βU(12)]−1 can be written as a sum of two terms:

(4) $$U\left(12\right)=U_{hs}\left(r\right)+U_{as}\left(12\right),\;\;\;\;f\left(12\right)=f_{hs}\left(r\right)+F_{as}\left(12\right),$$

where 1 and 2 denote the position and orientation of particles 1 and 2, $U_{hs}\left(r\right)$ is the potential of the hard spheres of size σ, $f_{hs}\left(r\right)=\exp\left[-\beta U_{hs}\left(r\right)\right]-1$, $F_{as}\left(12\right)=\exp\left[-\beta U_{hs}\left(r\right)\right]f_{as}\left(12\right)$, $f_{as}\left(12\right)=\exp\left[-\beta U_{as}\left(12\right)\right]-1$, and

(5) $$U_{as}\left(12\right)=\left\{\begin{array}{c} -\epsilon_{w}\;\;\mathrm{for}\;\;x\left(12\right)<a_{w}\\ \;\;\;0\;\;\mathrm{for}\;\;x\left(12\right)>a_{w} \end{array}\right.,$$

where x(12) is the distance between the square-well sites (see Figure 2), $\epsilon_{w}$ (>0) is the depth of the square-well potential, and $a_{w}$ is the square-well potential width, which is chosen to be small enough to ensure that not more than one bond per site can be formed. The multidensity version of the virial expansion is represented by the infinite series of terms (integrals), which involve the densities of the particles in different bonding states and are built on $f_{hs}$-bonds and $F_{as}$-bonds. In the two-density versions of the virial expansions for the Helmholtz free energy *A* and density ρ, we neglect the terms containing more than one association bond $F_{as}\left(12\right)$; as such, we finally have

(6) $$\frac{\beta A}{N}=\frac{\beta A_{hs}}{N}+\ln\left(\frac{\rho_{0}}{\rho }\right)-\frac{1}{2}\left(\frac{\rho_{0}}{\rho }\right)+\frac{1}{2}$$

and

(7) $$\rho =\rho_{0}+4\pi \rho_{0}^{2}\Delta_{as},$$

where $A_{hs}$ is the Helmholtz free energy of the hard-sphere fluid, $\rho_{0}$ is the density of non-bonded (free) particles, $\Delta_{as}=\int_{\sigma }^{\sigma +a_{w}}r^{2}\langle f_{as}\left(12\right)\rangle g_{hs}\left(r\right)dr$, $g_{hs}\left(r\right)$ is the hard-sphere radial distribution function, and 〈…〉 denotes orientational averaging. Thus, for the model in question, we have the following [113]:

(8) $$\langle f_{as}\left(12\right)\rangle =\frac{\exp\left(\beta \epsilon_{w}\right)}{6\sigma^{2}r}{\left(a_{w}+1-r\right)}^{2}\left(2a_{w}-1+r\right).$$

The solution of the quadratic Equation (7) for $\rho_{0}$ is used to calculate the Helmholtz free energy (Equation (6)). All of the other thermodynamic properties can be calculated using the Helmholtz free energy and standard thermodynamic relations. This theory has been extended in a number of different ways, in particular to treat network-forming fluids represented by models with several attractive sites (patches). Models of this type can be used to describe the effects of association and phase behavior in protein solutions. For the sake of illustration, we present expressions for the Helmholtz free energy of the model used to study the phase behavior of a solution of lysozyme [103]. This model is very similar to the one discussed above, being represented by a hard-sphere fluid with $n_{s}$ off-center square-well sites placed on the surface. It is assumed that these sites are equivalent and that pair interaction between any two sites located on different particles is described by the expression in (5). According to the TPT, the Helmholtz free energy of the model is

(9) $$\frac{\beta A}{N}=\frac{\beta A_{hs}}{N}+n_{s}\left(\ln X-\frac{1}{2}X+\frac{1}{2}\right),$$

where *X* is the fraction of particles with one arbitrarily chosen non-bonded site and *X* satisfies the following quadratic equation:

(10) $$4\pi n_{s}\rho \Delta_{as}X^{2}+X-1=0.$$

Note that for $n_{s}=1$, both of these equations reduce to Equations (6) and (7), which describe dimerization.

## 6. The Wertheim Theory Can Model Globular Protein Association

The theory outlined above has been used to model protein–protein interactions. We have calculated the properties of lysozyme solutions including the salt specific effects [103,114], analyzed crystallin mixtures [115], and modeled dumbbell-shaped proteins [116]. A useful experimental property is the cloud point temperature, $T_{cloud}$. This is the temperature below which a solution undergoes a liquid–liquid phase separation. By measuring the cloud point temperatures of different solutions, the liquid–liquid phase equilibrium curve can be constructed; see for example [117,118,119,120].

**Figure 3:**
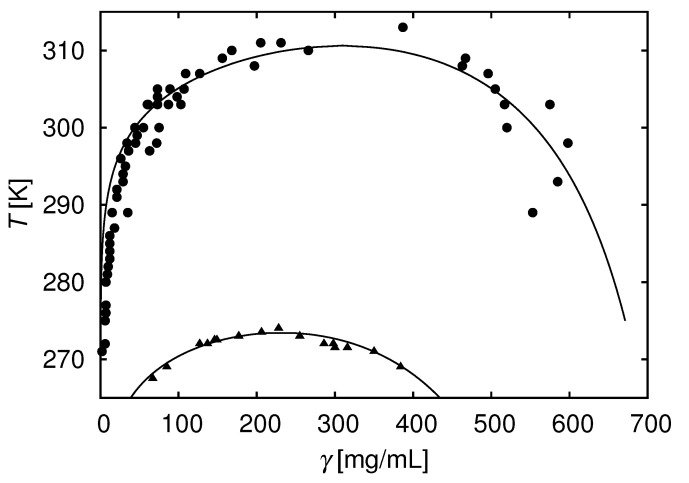
Liquid–liquid phase equilibria: theory and experiment. Top: γ IIIa-crystallin. Bottom: lysozyme. Solid curves are calculated from the model; see [103] for details. Experimental data shown by symbols taken from Taratuta [117] (upper symbols) and Broide [118] (lower symbols). Reprinted by permission from [103].

In [103], we analyzed experimental data published by Taratuta [117] and for another protein by Broide [118]. We modeled protein solutions as a single-component system of hard-sphere protein molecules embedded in an implicit solvent composed of water, buffer, and various simple salts. Each protein had a number of square-well attractive sites located on its surface (see Figure 2). Figure 3 compares the model results to experimental phase diagrams of two proteins from Figure 2 of [103]). The parameters required for the model are the diameter of the protein, strength of binding $\epsilon_{w}$, number of sites *M*, and range of the interaction $a_{w}$. We assumed that the short-range forces are dominant close to the phase transition, and chose $a_{w}$ = 0.18 nm, which is roughly length of the hydrogen bond (see Equation (5)). This provides a good agreement with experiments as well as with the second virial coefficients and osmotic compressibilities [103].

**Figure 4:**
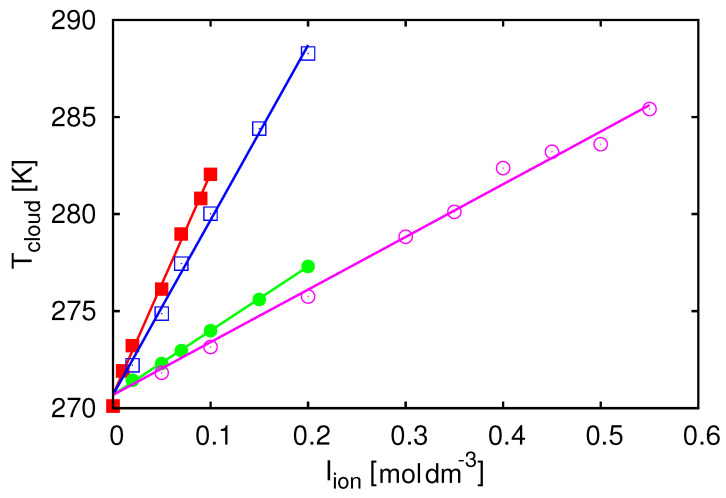
Experimental data modulation of protein interactions by salts in lysozyme solutions. $T_{cloud}$ for lysozyme as a function of ionic strength of the added alkali-halide salts $I_{ion}$; the symbols denote experimental data [117]. The lines are the results of Equation (12) from [103] (from top to bottom: KBr, NaBr, KCl, and NaCl salts). Reprinted by permission from [103].

This model helps to interpret how protein–protein interactions are modulated by added alkali–halide salts such as NaCl, KCl, NaBr, and KBr [117]. In principle, salts and excipients can be treated explicitly as distinct molecular species within the Wertheim theory. Instead, we have done something much simpler here, while continuing to provide useful insights. Specifically, we take experimental data on how $T_{cloud}$ depends on the ionic strength of the electrolyte $I_{ion}$ for different ions (Figure 4) and then fit these data using straight lines:

(11) $$\epsilon_{w}/k_{B}=aI_{ion}+b$$

where *a* and *b* are line fit constants. The increase in the cloud point temperature $T_{cloud}$ indicates a decrease in the stability of the system. The results show that bromides destabilize the solution more than chlorides and anions do so more than cations (the experiment was performed under conditions where the net charge was positive). This approach makes it possible to assess the nature of salt effects.

**Figure 5:**
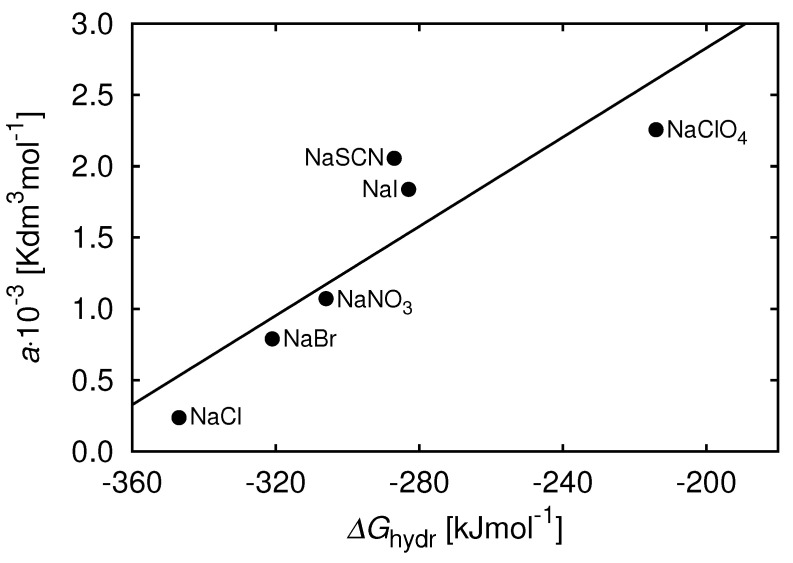
The ions that bind most weakly to water most strongly affect the protein-protein attraction. Correlation between slope *a* in Equation (12) [103] and the Gibbs free energy of hydration $\Delta G_{hydr}$ for the corresponding anions. Reprinted by permission from [103].

Why do bromides destabilize the solution more than chlorides? To address this question, we can compare the slope quantity *a* from Equation (11) above to the Gibbs free energy ($\Delta G_{\mathrm{hydr}}$) of hydration for the ion, which provides the affinity of the ion when binding solvent water molecules. Figure 5 along with the $\Delta G_{\mathrm{hydr}}$ data from [120] shows how these quantities relate. The ordering follows the inverse Hofmeister series [120]. Figure 5 shows that those ions that most easily release their bound water are the ones that most readily bind to the proteins and most strongly affect the protein-protein attraction.

The fact that cloud point temperature data can provide valuable insights into salt-specific effects was further confirmed in [114], where we applied such an analysis to our own measurements performed on lysozyme solutions in mixture with more low-molecular salts. In the same article, we demonstrated how this theory can predict liquid–liquid phase diagrams under conditions where no phase separation experiments have yet been carried out, using only the cloud point temperature measurements at low protein concentrations as input. These are very convincing examples that indicate how theoretical analysis can contribute to better understanding of protein solutions.

Thus far, we have described how the Wertheim approach can be used for proteins of simple shapes, such as spheres. Below, we consider antibody molecules, which have more complex shapes.

## 7. Structure–Property Relations of Antibodies: The Seven-Bead Wertheim Model

The structure, size, and flexibility of antibody molecules are reflected in their properties. Experimental data indicate a sharp increase of the viscosity around protein concentrations approaching 100 mg/mL; see for example [8]. This is mostly due to aggregate formation, and as such concentrations above this value cannot be recommended for medical applications. Another property in which antibodies differ from the globular proteins reviewed here is in the liquid–liquid phase diagram. The critical concentration in the case of antibody solutions is much smaller than for globular proteins, while the width of the two-phase region is larger relative to the critical concentration (see Figure 4 in [121]).

**Figure 6:**
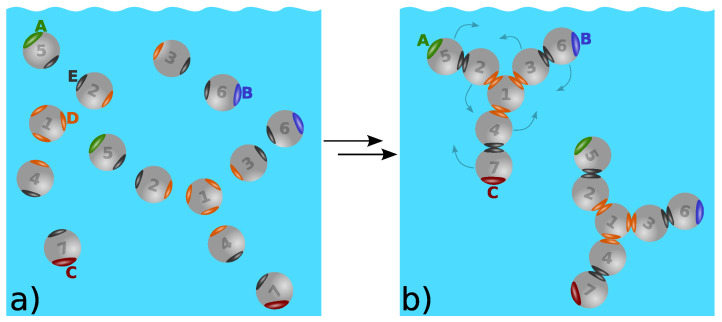
(**a**,**b**) Seven-bead model molecules. Each Y-shaped molecule first assembles from seven individual beads via strong forces, which act only between the sticky spots of the same color. Next, these molecules associate into non-covalent clusters. A and B denote the Fab fragments (the region of the antibody that binds to antigens), while C denotes the Fc arm (called the fragment crystallizable region, which interacts with the cell surface receptors). Figure reprinted by permission from [56]. Copyright Elsevier (2017).

While there have been other interesting approaches to modeling antibodies [8], the one we describe here applies the Wertheim theory to complex protein structures. The seven-bead model and Wertheim’s theory have allowed us to analyze many valuable measurements. Figure 6 shows how Y-shaped antibody molecules can be represented as seven coarse-grained beads [56]. In a first step, seven spherical beads associate into a Y shape, representing the covalent interactions. Next, these Y-shaped molecules populate a solution and are able to associate in structure-specific ways depending on the locations and orientations of the bead binding sites.

In principle, Wertheim’s theory can be formulated for any number of sticky sites and any number of the bead species. The version of the theory used here is obtained as a limiting case of infinitely strong bonding interactions connecting beads to form a particular shape (see Figure 6). It is possible to model molecules of other shapes, i.e., fused spheres, linear polymers, V-shape molecules, etc. The theory has been described in detail in a number of previous publications [56,116,122,123,124].

**Figure 7:**
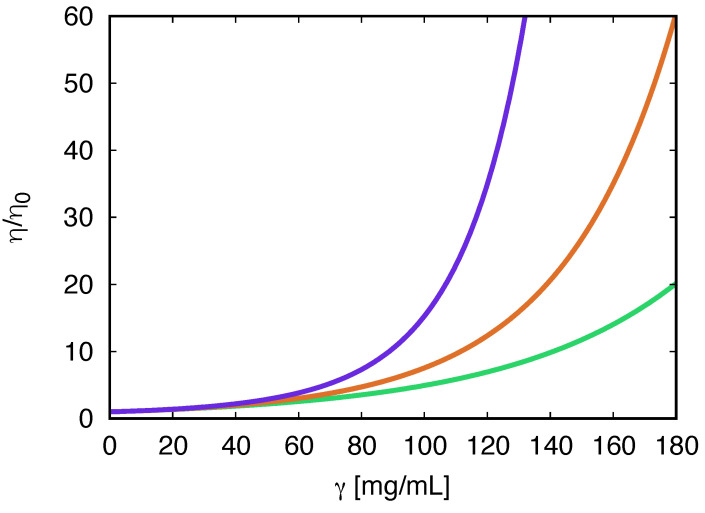
Relative viscosity $\eta /\eta_{0}$ as a function of the protein concentration [56]. From bottom to top: (i) model of bispecific antibodies, green curve; (ii) symmetric Fab–Fab model of antibodies, red curve; and (iii) model of interacting Fab–Fc terminals, blue curve. For more details, see the original paper. Figure reprinted by permission from [56]. Copyright Elsevier (2017).

This theory provides several interesting results. One is that aggregation can be efficiently controlled by modifying the antibody itself, not only by additives dissolved in the antibody solution. We interpret the results shown in Figure 7 of this work as follows: (i) the antibodies having two identical Fab arms form linear chains, causing intermediate viscosities (the red curve); (ii) bispecific antibodies having different Fab arms can cause low viscosity of the solution in cases involving dimerization (the green curve); (iii) arm-to-Fc binding, which can be caused, for example, by charge complementarity, allows for three binding partners, leading to networks and high viscosities (the blue curve).

**Figure 8:**
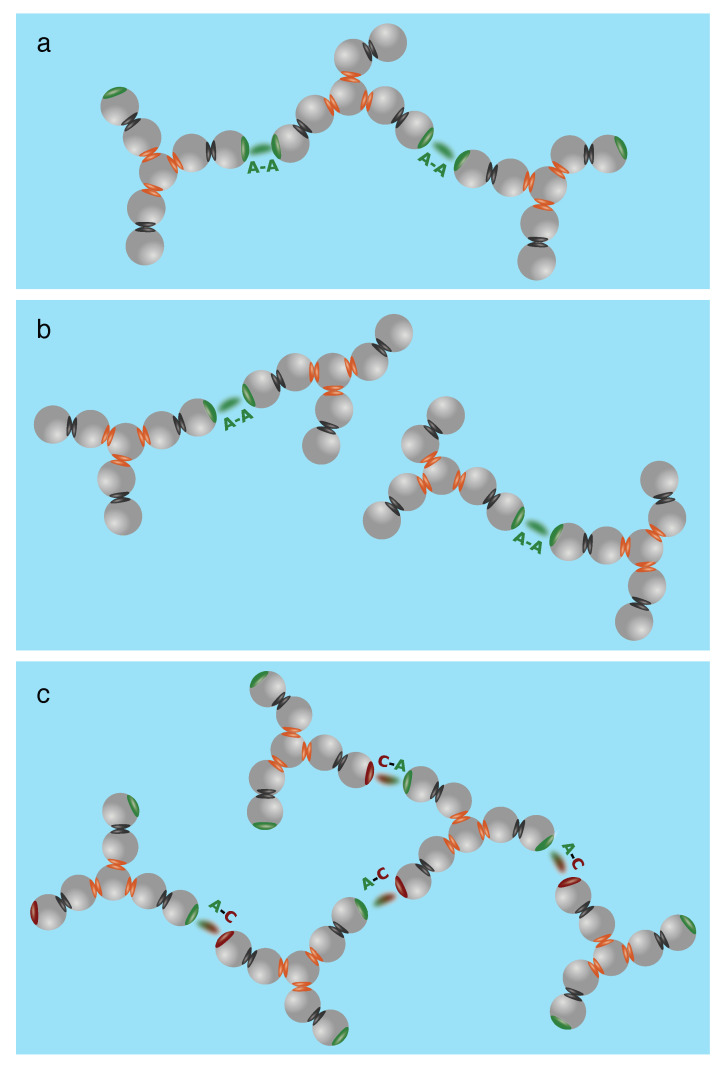
Three types of antibody clustering studied in this work. The types of antibody clustering studied in this work are: (**a**) monospecific two-arm binding; (**b**) bispecific single-arm binding; and (**c**) arms-to-Fc binding. Figure 8 reprinted by permission from [56]. Copyright Elsevier (2017).

The results of the calculations presented in Figure 7 are graphically illustrated in Figure 8. Panel (a) represents the case in which linear chains are formed (the red line in Figure 7), while panel (b) shows the low-viscosity case where only pairs exist (the green curve in Figure 7) and panel (c) illustrates the high viscosity case, represented by the blue curve in Figure 7. In general, it can be said that the viscosity depends on how many neighbors the molecule can link. The possibility of such linking is what has to be prevented in the process of antibody design.

In view of importance of the biomolecular chemistry taking place in liquid-phase separated compartments [125,126,127], several of our studies have been dedicated to modeling such phase separations. Protein droplets (membraneless organelles) formed via liquid–liquid phase separation are currently the center of much interest on the part of many researchers; however, it is not within the scope of this contribution to review the vast literature in this area of protein science. In the following, we focus on a few examples for which the available experimental data are suitable as a subject of this analysis. Note that such data are not in abundance; on the contrary, there is limited availability on the part of the relevant protein drugs, meaning that experiments are often incomplete and not always well documented.

**Figure 9:**
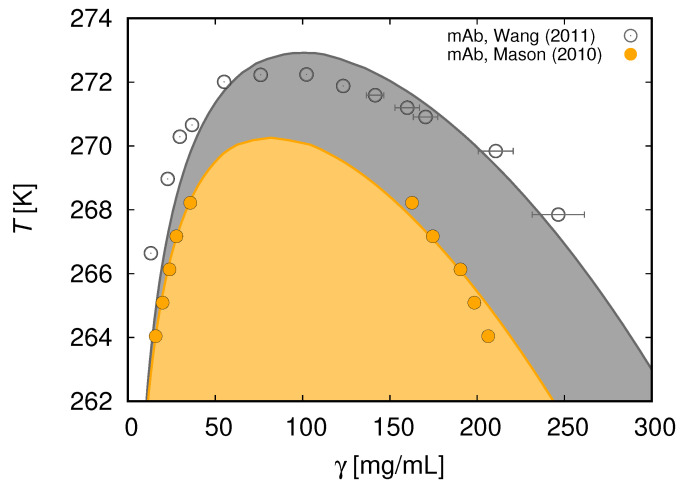
The liquid-liquid phase equilibria of mAb solutions. Temperature *T* vs. mAb concentration: calculated (full line) and symbols (experimental data) [121,128]. The interaction between sites A, B, and C is modeled using the short-range square-well attraction. The two-phase region is indicated by the colored area; for more details, see [123]. Reprinted with permission from [123]. Copyright 2018 American Chemical Society.

In Figure 9, we show that the seven-bead model provides good fits of the liquid–liquid phase equilibria for two different antibody solutions [123]. In our analysis, we have assumed that the A–B, A–C, and B–C interactions are attractive, while for the A–A, B–B, and C–C interaction sites the attraction was set to zero. Note that in this case A and B again denote the Fab arms, while C stands for the Fc (crystallizable segment) arm of the antibody. In agreement with measurements, our calculation finds the critical concentration of antibodies to be lower than for globular proteins. An alternative choice in which A–A, B–B, A–C, and B–C pair interactions are dominant has been suggested by Calero-Rubio and colleagues [17]. Their article is based on the computer simulation of a more sophisticated model than ours. Note that the choice of interaction parameters depends on the nature of the antibody being studied.

## 8. Additional Components Can Modulate Protein Associations

Protein assemblies can be stabilized or destabilized by adding other molecular components. One example is the excipients and salts that are added to biological antibody drug formulations to keep the proteins folded and disaggregated. Another example is in protein assemblies called membraneless organelles, which contain multiple macromolecular components, i.e., other proteins, RNAs, and/or other macromolecules [3,38,129,130]. The Wertheim approach is readily generalized for multiple components, at least for the simple situations explored thus far.

One case we studied was mixtures of β and γ lens crystallin proteins [59]. We treated the proteins as being dumbbell-shaped with different interaction energies, and calculated the equilibrium phase diagrams [115,116]). The analysis yields good agreement with experimentally determined quantities, such as the phase diagram, isotherms, and even the tie-lines, which are very sensitive to model parameters. The calculation provides results for pure protein β, for which no direct measurements are available. The calculation of the critical temperature is in good agreement with the value suggested from extrapolation of experimental data; the added component protein decreases the critical temperature relative to the pure system.

In a second example, we used the theory [131] to model a human monoclonal antibody in mixtures with human serum albumin (HSA) [121]). We treated the antibodies using the seven-bead model and HSA as hard spheres with attractive sites to bind other molecules. The modeling is consistent with experiments which show a significant decrease of the critical temperature in presence of HSA and the preferential partitioning of HSA into the antibody-rich phase.

In yet another example [122], we applied the theory to monoclonal antibody solutions in the presence of the polymer polyethylene glycol (PEG) [61]. The polymer was treated as a chain of linked hard spheres. The polymer simply introduces an entropic contributor to the liquid–liquid phase separation, and, as above, decreases the critical temperature.

We additionally explored how monoclonal antibody solutions can be stabilized in the presence of excipients [132]. For excipients with an affinity to bind to antibody sites, their binding weakens the alternative protein–protein binding, which reduces protein association, thereby reducing the viscosities.

## 9. Crowding Can Alter Protein–Protein Association

Statistical mechanics-based theories can be used to explore the effects of constraints and environments. The insides of biological cells are regarded as lcrowded because of the presence of a high density of molecular obstacles [133]. We have studied antibodies in two situations: one in which crowding agents are inert and their volume is simply excluded from occupancy by the antibodies [134], and one in which the obstacles are attractive for the antibodies [135]. In both cases, the obstacles are fixed (i.e., they do not move) in random positions. The antibodies are modeled as seven-bead particles which can interact with each other and are otherwise constrained by the obstacles [134,135]. In the case of particles that simply occupy volume, the presence of obstacles merely restricts the space available to antibodies.

The theory used here is somewhat more complicated. This is because our system is not a binary solution; in our case, one component (the proteins) is mobile and the other (the obstacles) is fixed. This kind of “partly quenched” system has to be treated differently than a solution. Following previous works (see [134,136] and references therein), we used a combination of the scaled particle theory, Wertheim’s thermodynamic perturbation theory, and the Flory–Stockmayer theory to calculate various measurable properties, such as the liquid–liquid phase separation, percolating region, and others. The conclusion that we reached is that hard-sphere obstacles strongly decrease the critical density and marginally decrease the critical temperature. As expected, confinement enhances clustering, and the strength of this effect depends on the packing fraction of obstacles $\eta_{0}$. We are not aware of any computer simulations or experimental data performed on such systems; however, the results are in qualitative agreement with our recent experimental observations on liquid–liquid phase separation of bovine serum albumin in the presence of polyethylene glycol [137].

**Figure 10:**
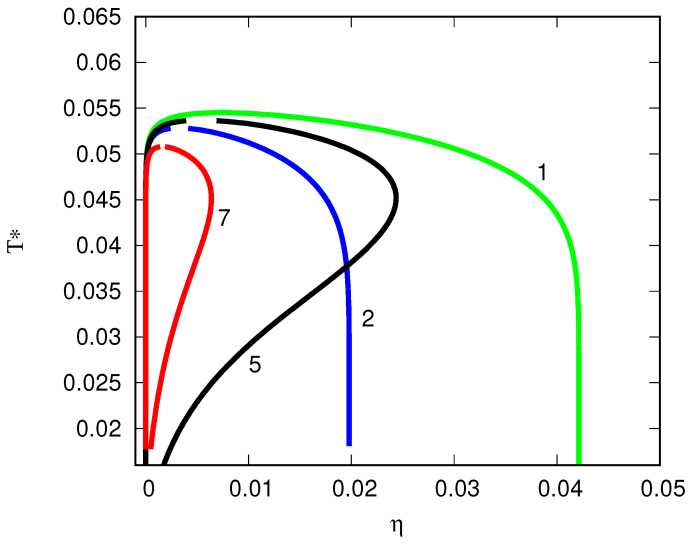
Liquid-liquid phase diagrams of antibody solutions in the presence of attractive obstacles. Phase diagrams $T^{*}$ vs. $\eta =\pi \rho_{1}^{*}/6$ coordinate frame for model of monoclonal antibodies in Yukawa hard-sphere porous media at bonding distance $0.05\sigma_{1}$ and for $\epsilon_{AA}^{\left(as\right)}=\epsilon_{BB}^{\left(as\right)}=\epsilon_{AB}^{\left(as\right)}=\epsilon$, $\epsilon_{CC}^{\left(as\right)}=0$, $\epsilon_{AC}^{\left(as\right)}=\epsilon_{BC}^{\left(as\right)}=\epsilon$, obstacles packing fraction $\eta_{0}=0.1$, and different strengths of the Yukawa interaction: $\epsilon_{Y}=0$ (blue (2) line), $\epsilon_{Y}=0.06\epsilon$ (black (5) line), and $\epsilon_{Y}=0.1\epsilon$ (red (7) line). The green (1) line denotes the result for the neat fluid (no obstacles present, $\eta_{0}=0$). Reproduced in part from Ref. [135]. Reproduced with permission of the Royal Society of Chemistry.

We additionally studied antibodies in the presence of attractive obstacles [135]. Here, the antibodies interact through Fab–Fab and Fab–Fc attractive interactions; we assumed that the antibodies interact with the obstacles through a Yukawa attractive potential. The value of this type of statistical mechanical modeling lies in its ability to explore physics that go beyond MD and to make predictions of as yet unexplored experimental behaviors. We computed the liquid–liquid phase behavior, cluster size distributions, and second virial coefficients as functions of the protein properties, obstacle properties, and obstacle–protein interactions. Adding an attractive potential to the obstacle–antibody interaction first increases the width of the phase diagram (T*-ρ*) envelope, then, upon further strengthening of the attractions to the obstacles, shrinks the width. For more details, see Figure 2 in the original work [135]. The general behavior of such systems is illustrated here in Figure 10. The green line (1) describes the phase diagram for a neat antibody solution and the blue line (2) the one in the presence of the hard-sphere particles. At a certain point, depending on the obstacle–antibody attraction $\epsilon_{Y}$, a situation is observed in which two different temperatures have the same fluid density; this is called the re-entry point.

The re-entry points shown here have been found before in the “patchy colloid” systems examined by Sciortino and coworkers and documented in [138]; additionally, see [95,139]. It needs to be stressed, however, that in our case the underlying physics are different from those identified in Sciortino’s papers. Here, the protein–obstacle attraction causes strongly nonuniform antibody distributions centered on the immobile obstacles, suppressing the phase separation.

Considering the implications of this, first, cells may leverage and control these physical interactions [125,127,129,140]. Membraneless organelles may form and disintegrate at various places in the cell or various times in its lifecycle [125,126,140]. Second, this statistical mechanical modeling can be useful in developing formulations of of biological drugs while taking into account the temperature, excipients, polymer stabilizers, containers, and confinement (for example) as well as the protein properties.

## 10. Limitations and Challenges of Statistical Mechanical Modeling

The statistical physics model described here has both advantages and disadvantages. While molecular dynamics simulations can capture more atomistic details and do not require free parameters, statistical mechanical theories can handle more than just a few protein molecules at a time and can sample the full configuration space much more broadly; as such, they can provide viable predictions of the phase equilibria, viscosities, second virial coefficients, and others. The Wertheim approach allows a level of granularity when treating different proteins as having different structures, rather than just as spheres.

Formally, any macromolecule that can be depicted as a collection of the spherically symmetric monomers is amenable to treatment using Wertheim’s approach; however, the accuracy of the results depends on the number of the monomers and the number of bonds holding the the monomers together. In general, the smaller the number of monomers, the higher the accuracy; on the other hand, this requires making choices when modeling the formation of macromolecular clusters. First, it must be determined where and how many binding sites there are and what their affinities are. For seven-bead antibody modeling, it is natural to assume binding at the Fab or Fc fragments. Second, we have only treated the short-ranged interactions here. For this, we are required to obey Equation (5) for the range of interaction; it is necessary that the square-well potential width be small enough to ensure that not more than one bond per site can be formed. We did not consider cases where the Coulomb forces are important and the choice of buffer, electrolyte nature, and its concentration play a role [9,19,141]. The theory itself can in principle be extended to (approximately) treat electrostatics [142] as well as to analyze salt-specific effects and Hofmeister ordering in protein solutions [143]; however, we have not done this here.

As a given macroscopic property can typically be reproduced in multiple ways with different choices of microscopic parameters, statistical mechanical theories can be regarded as a sort of engineering tool to be combined with other insights, experimental data, or atomistic simulations that constrain the parameter space. For example, for spherical proteins, simply knowing the pairwise protein–protein affinity (either from measurements of the dilute solution or from molecular simulations) is sufficient, when used with the Wertheim theory, to predict multi-body properties such as viscosities of dense solutions or liquid–liquid phase equilibria.

In general, coarse-grained multi-bead models can be quite useful in this way. The twelve-bead MD simulations by Truskett et al. harness scattering data to predict macro-scale properties ([28,29,30,31,32]). Similarly, Roberts et al. combined MD simulations with experimental second virial coefficient observations ([17,74,75,76,77]).

## 11. Conclusions

Protein–protein interactions are often strong, leading to multi-body clusters and solutions that have high viscosities and liquid phase separations. Modeling such solutions has been difficult in the past, as molecular simulations encounter challenges when handling multiple proteins and as older statistical mechanics-based models are limited to treating proteins as spheres without any structure-property relationships. Here, we have reviewed the Wertheim approach, which can handle multi-body interactions of proteins (although still coarse-grained) with more detailed structures. This approach promises the ability to ultimately handle additional components, such as excipients and salts, and obtain the binding parameters from fine-grained molecular simulations, completing the link between atomistic modeling and macroscopic solution behaviors.

## Figures and Tables

**Table 1:** Early work on statistical mechanical models and experiments.

When	What	Who
1990’s	DLVO theories	Prausnitz
1990’s	Liquid phase diagrams	Benedek
1994	Crystalization, B${}_{22}$	George and Wilson
1999	Wertheim’s theory	Sear
2000’s	Protein stability	Randolph, Carpenter, Shire
2000’s	Salt specific effects	Ninham, Kunz, Tavares
2015	Aggregation	Kerwin, Roberts
2015	Molecular structure	Schmitt, Kerwin, Zhou
